# Research Progress in RNA-Binding Proteins

**DOI:** 10.3390/ijms24010058

**Published:** 2022-12-21

**Authors:** Alexandre Smirnov

**Affiliations:** 1 UMR7156—Génétique Moléculaire, Génomique, Microbiologie (GMGM), University of Strasbourg, CNRS, 67000 Strasbourg, France; alexandresmirnov@unistra.fr; 2 University of Strasbourg Institute for Advanced Study (USIAS), University of Strasbourg, 67000 Strasbourg, France

RNA-binding proteins are everywhere and accompany RNA molecules at every stage of their molecular life, from “birth” (transcription) through “growing up” (maturation), “active life” (molecular function) until “death” (turnover). This Special Issue offers insight into some of the most fascinating proteins of this group that function in various compartments of eukaryotic cells, under diverse circumstances, and play dissimilar, but invariably essential, roles (Figure 1).

The Special Issue first addresses transcription and its main actor, RNA polymerase, a term that groups a variety of unrelated enzymes that produce all cellular RNAs. Interestingly, in eukaryotic viruses of the Mononegavirales order, including the Ebola, measles, mumps, and rabies (RABV) viruses, the RNA-dependent RNA polymerase consists of two subunits, the large protein (L) and the phosphoprotein (P). The L protein is the catalytic subunit required for RNA synthesis, capping, and polyadenylation. The P protein is its obligatory cofactor, which is also usually responsible for the stability and solubility of the polymerase. Viruses often rely on abundant host-cell proteins to carry out their functions. In this case, the formation of an active viral polymerase complex depends on the host-cell chaperone Hsp90, which promotes the folding and stability of the L protein. However, RABV and other lyssaviruses have been considered as an exception from this standard scenario, as in their case, Hsp90 has been proposed to stabilise the P protein instead [10]. In this Special Issue, Dalidowska et al. argue that this might not be the case [9]. The authors find, surprisingly, that the RABV L and P proteins do not require Hsp90 for intracellular accumulation, nor does the P protein seem to be necessary for L protein solubility. By contrast, Hsp90 activity seems to be essential for the assembly of the functional RNA polymerase holoenzyme during infection, which may be a new mechanistic peculiarity of RABV.

Even before transcription ends, RNA molecules enter the processing line, which is managed by an array of protein and ribonucleoprotein biogenesis factors that help to fold, modify, and cleave precursor transcripts, and hand the resulting mature RNAs over to the downstream proteins for transport and the realisation of their molecular functions. Among these factors, ribonucleases hold a special place, and three papers in this Special Issue are dedicated to this class of enzymes [6,7,8].

One of the best-studied processing factors, responsible for the production of mature miRNAs and siRNAs, is the Dicer protein, involved in diverse RNA interference pathways in eukaryotes. Although a large number of papers have seemingly addressed all the biochemical, structural, and regulatory aspects of its function in a variety of models, quite a few areas remain understudied and do not cease to intrigue researchers. One such facet is the mysterious DUF283 domain, a domain of unknown function, as its name indicates, which had been found to possess ssRNA-binding and nucleic acid-annealing activities [11,12]. In a follow-up study published in this Special Issue, Szczepanska et al. hypothesised that this part of the protein might be responsible for the elusive RNA base-pairing ability of human Dicer [6]. They designed and purified human Dicer variants that lacked the DUF283 domain and assayed their activities in vitro and in vivo. While both of the tested mutant proteins could cleave model substrates, often as efficiently as full-length Dicer, they lost their RNA annealing activity, confirming the authors’ hypothesis. It remains to be established how exactly this unusual, but deeply conserved, biochemical function of Dicer proteins is implemented in cells and what its global significance is for post-transcriptional gene expression control.

While Dicer is one of key RNases in the cytosol, a number of remarkable nucleolytic enzymes have been identified and functionally characterised in the eukaryotic organelles of bacterial origin, mitochondria and chloroplasts. Partly inherited from their bacterial ancestors and partly invented de novo upon their ‘domestication’ by their eukaryotic hosts, these RNases often play essential roles in organellar gene expression and thereby critically contribute to their core functions—respiration and photosynthesis. A review article by Cartalas et al. in this Special Issue provides a thorough, systematic survey of the wide diversity of mitochondrial ribonucleases and covers various aspects of their biology, including phylogenetic distribution, structural and biochemical particularities, and established biological roles [8]. The authors not only included well-recognised enzymes, such as diverse RNases P and Z classes, but also a few less understood, sometimes controversial, yet biologically important proteins, such as FASTKD family factors [13], RNase T2 [14], and YBEY [15]. Understanding the functions and the molecular mechanisms of these enzymes constitutes one of the next significant challenges in the field of mitochondrial post-transcriptional regulation and beyond.

In the same vein, a comprehensive review by Falchi et al. tells about one of the most deeply conserved and functionally important RNases, polynucleotide phosphorylase (PNPase) [7]. Focusing on the human PNPase homologue, the authors discuss in great detail how PNPase expression is regulated, how this enzyme is structurally organised, how it binds and cleaves RNAs, and what roles it plays in our mitochondria (and eventually, in other cellular compartments).

With a continued focus on the eccentric world of organellar RNA biology, a review by Li et al. in this Special Issue explores the important class of pentatricopeptide repeat (PPR) proteins, which greatly expanded in eukaryotic mitochondria and plastids, especially in ‘green clades’ [5]. Many of them are RNases or accessory factors that specifically interact with target RNAs and participate in their processing, editing, splicing, stabilisation, or translation. The authors showcase their macroscopic, physiological roles and provide many examples of the extremely diverse molecular mechanisms used by PPR proteins in plants.

Interestingly, in many groups, including algae, plants, and mammals, quite a few PPR proteins have been incorporated into mitochondrial ribosomes as new, supernumerary structural components. A review by Scaltsoyiannes et al. in this Special Issue invites the reader to discover a fascinating gallery of mitochondria-specific proteins that ‘augmented’ the ribosomes of their bacterial ancestor, with 100+ novel polypeptides with diverse origins and structures [4]. Sometimes compensating for lost ribosomal RNA and often conferring new, mitochondria-specific functionalities linked to their unique evolutionary constraints and the very nature of the polypeptides they help to produce, these proteins show us how changeable the most conserved gene expression machineries may be, and how much new, unexpected biology one can discover by looking at less traditional and phylogenetically distant models.

Ribosomes are central but not the only players in translation. This process is especially complex in eukaryotes, which deploy a large array of RNA-binding proteins to modulate the efficiency of translation initiation by a variety of mechanisms, including mRNA modification. For example, it was shown that the wide-spread m^6^A modification in the 5′-UTRs of many messengers enables cap-independent translation under stress conditions [16]. In this Special Issue, Sakharov et al. confirm this earlier observation using in vitro translation and toeprinting assays [3]. Furthermore, they find that, while under normal conditions, the initiation and elongation on a methylated mRNA are less efficient, the presence of m^6^A in the 5′-UTR makes the initiation more resistant to the inhibition of the canonical cap-dependent scanning. This unexpected result suggests the existence of a non-canonical (probably ATP-independent and eIF3-mediated) initiation mechanism that is uniquely available to m^6^A-containing messenger RNAs, which guarantees their preferential translation under stress.

The m^6^A modification has its downsides. It destabilises the messenger through the interaction with YTHDF2 that transfers it to repressive condensates, such as P-bodies and stress granules (SGs) [17]. Two research papers in this Special Issue focus on SGs, their conserved components and some new, unexpected properties [1,2]. Stress granules typically form, as the name suggests, under stress conditions, which activate eIF2α kinases that phosphorylate, and thereby inactivate, the initiation factor eIF2. This results in the accumulation of stalled translation initiation complexes, which phase-separate into SG condensates. An essential player in SG formation, and their diagnostic marker, is the Ras-GAP SH3 domain-binding protein (G3BP), which in humans is encoded by two redundant genes. As it is often the case with plants, the *A. thaliana* genome encodes as many as seven G3BP homologues, raising questions of their respective molecular roles and their involvement in SG formation. An elegant study by Reuper et al. in this Special Issue leverages trans-kingdom functional complementation to single out two plant G3BP-like proteins that can rescue sodium arsenite-induced SG formation in human cells that lack both native G3BP homologues [1]. Even more strikingly, domain swaps, in which the RG/RGG domains of the plant proteins were grafted on the human G3BP-1 scaffold, all turned out to be proficient in SG formation. This surprising finding suggests that not only the presence of the RG/RGG domain, but also its proper positioning with respect to the other domains of G3BP define its ability to drive phase separation during SG formation.

SGs do not always form via the above-described canonical mechanism. For example, the treatment of yeast and mammalian cells with inhibitors of the respiratory chain results in the formation of unusual SGs. In this Special Issue, Eiermann et al. study in detail the SGs induced by sodium azide, a potent inhibitor of the mitochondrial complex IV [2]. The authors show that sodium azide stalls translation and triggers the formation of very small SGs in mammalian cells. Surprisingly, their data indicate that this happens by a mechanism independent of eIF2α phosphorylation. The formation of these tiny SGs, however, seems to be linked to global ATP depletion caused by inhibited respiration. It appears that SGs are much more heterogeneous, both composition-wise and mechanistically, than we used to think. These omnipresent condensates certainly conceal many more surprises awaiting discovery.

## Figures and Tables

**Figure 1:**
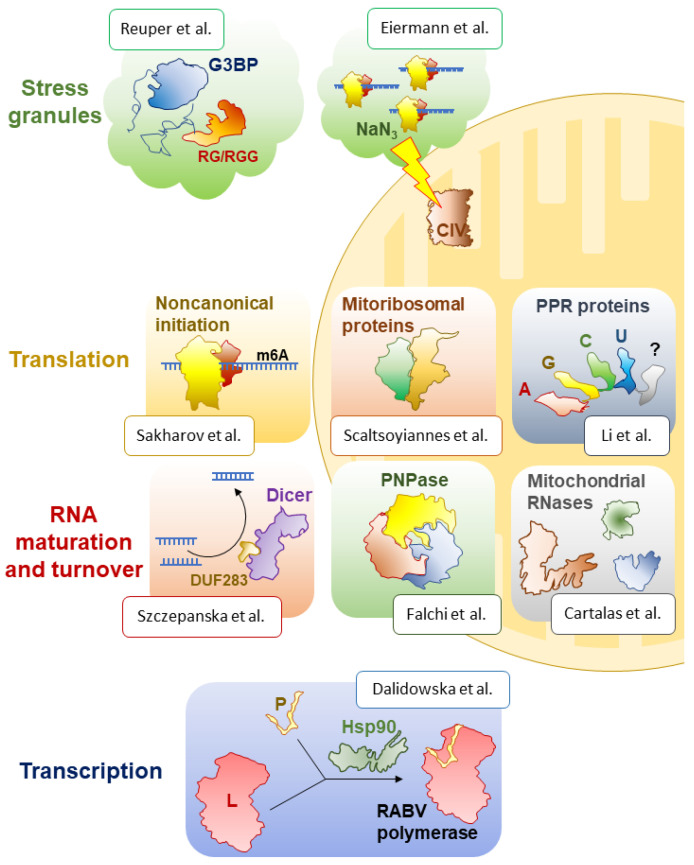
Overview of the contents of the Special Issue “*Research progress in RNA-binding proteins*” [1,2,3,4,5,6,7,8,9].

## References

[1] Reuper H., Götte B., Williams L., Tan T.J.C., McInerney G.M., Panas M.D., Krenz B. (2021). Arabidopsis Thaliana G3BP Ortholog Rescues Mammalian Stress Granule Phenotype across Kingdoms. Int. J. Mol. Sci..

[2] Eiermann N., Stoecklin G., Jovanovic B. (2022). Mitochondrial Inhibition by Sodium Azide Induces Assembly of EIF2α Phosphorylation-Independent Stress Granules in Mammalian Cells. Int. J. Mol. Sci..

[3] Sakharov P.A., Smolin E.A., Lyabin D.N., Agalarov S.C. (2021). ATP-Independent Initiation during Cap-Independent Translation of M6A-Modified MRNA. Int. J. Mol. Sci..

[4] Scaltsoyiannes V., Corre N., Waltz F., Giegé P. (2022). Types and Functions of Mitoribosome-Specific Ribosomal Proteins across Eukaryotes. Int. J. Mol. Sci..

[5] Li X., Sun M., Liu S., Teng Q., Li S., Jiang Y. (2021). Functions of PPR Proteins in Plant Growth and Development. Int. J. Mol. Sci..

[6] Szczepanska A., Wojnicka M., Kurzynska-Kokorniak A. (2021). The Significance of the DUF283 Domain for the Activity of Human Ribonuclease Dicer. Int. J. Mol. Sci..

[7] Falchi F.A., Pizzoccheri R., Briani F. (2022). Activity and Function in Human Cells of the Evolutionary Conserved Exonuclease Polynucleotide Phosphorylase. Int. J. Mol. Sci..

[8] Cartalas J., Coudray L., Gobert A. (2022). How RNases Shape Mitochondrial Transcriptomes. Int. J. Mol. Sci..

[9] Dalidowska I., Orlowska A., Smreczak M., Bieganowski P. (2022). Hsp90 Activity Is Necessary for the Maturation of Rabies Virus Polymerase. Int. J. Mol. Sci..

[10] Xu Y., Liu F., Liu J., Wang D., Yan Y., Ji S., Zan J., Zhou J. (2016). The Co-Chaperone Cdc37 Regulates the Rabies Virus Phosphoprotein Stability by Targeting to Hsp90AA1 Machinery. Sci. Rep..

[11] Kurzynska-Kokorniak A., Pokornowska M., Koralewska N., Hoffmann W., Bienkowska-Szewczyk K., Figlerowicz M. (2016). Revealing a New Activity of the Human Dicer DUF283 Domain in vitro. Sci. Rep..

[12] Pokornowska M., Milewski M.C., Ciechanowska K., Szczepańska A., Wojnicka M., Radogostowicz Z., Figlerowicz M., Kurzynska-Kokorniak A. (2020). The RNA-RNA Base Pairing Potential of Human Dicer and Ago2 Proteins. Cell. Mol. Life Sci..

[13] Jourdain A.A., Popow J., de la Fuente M.A., Martinou J.-C., Anderson P., Simarro M. (2017). The FASTK Family of Proteins: Emerging Regulators of Mitochondrial RNA Biology. Nucleic Acids Res..

[14] Liu P., Huang J., Zheng Q., Xie L., Lu X., Jin J., Wang G. (2017). Mammalian Mitochondrial RNAs Are Degraded in the Mitochondrial Intermembrane Space by RNASET2. Protein Cell.

[15] Liao Z., Schelcher C., Smirnov A. (2021). YbeY, Éminence Grise of Ribosome Biogenesis. Biochem. Soc. Trans..

[16] Meyer K.D., Patil D.P., Zhou J., Zinoviev A., Skabkin M.A., Elemento O., Pestova T.V., Qian S.-B., Jaffrey S.R. (2015). 5’ UTR m(6)A Promotes Cap-Independent Translation. Cell.

[17] Wang X., Lu Z., Gomez A., Hon G.C., Yue Y., Han D., Fu Y., Parisien M., Dai Q., Jia G. (2014). N6-Methyladenosine-Dependent Regulation of Messenger RNA Stability. Nature.

